# Synthesis of *C*-Arylnucleoside Analogues

**DOI:** 10.3390/molecules20034967

**Published:** 2015-03-18

**Authors:** Christophe Len, Gérald Enderlin

**Affiliations:** 1 Sorbonne Universités, Université de Technologie de Compiègne, Ecole Supérieure de Chimie Organique et Minérale, Transformations Intégrées de la Matière Renouvelable, Centre de Recherche Royallieu, CS 60319, Compiègne cedex F-60203, France; E-Mail: g.enderlin@escom.fr; 2 Department of Chemistry, University of Hull, Hull HU6 7RX, UK

**Keywords:** *C*-arylnucleoside, nucleoside, carbohydrate, total synthesis, asymmetric catalysis

## Abstract

Modified nucleoside analogues are of great biological importance as antiviral and antitumoral agents. There is special interest in the preparation of *C*-aryl nucleosides with an aromatic ring in different positions of the glycone for their biological activity. Different chemical synthesis strategies for these targets are described in this review.

## 1. Introduction

Nucleoside analogues have shown high effectiveness as antiviral and antitumoral agents. In order to improve the pharmacologic activity, a variety of functionalities have been introduced into either the ribose moiety [1,2,3,4] or the heterocyclic moiety [4,5], particularly an aromatic core. This review is focused on the synthesis of *C*-aryl nucleoside analogues having *C*-*C* bonds between an aryl core and the glycone moiety. The particular *C*-*C* bond formations covered in this review are those in positions 1', 2', 3', 4' and 5' of the ribose ring. The well-known *C*-nucleosides in which the anomeric bond has been replaced by a *C*-*C* bond have been the focus of recent reviews [6,7] and are therefore not included in this review. In this regards, this review has been arranged to describe the different methodologies for the formation of *C*-aryl bond according to the type of organic reaction involved: addition to a carbonyl group, *C*-*C* cross-coupling, addition to epoxides and cyclization. One special section is dedicated to the formation of the glycone ring starting from an aromatic core.

## 2. Addition of an Aromatic Ring to a Carbonyl Group

Introduction of an aromatic core can occur via the attack of organometallic reagents such as a lithium, magnesium, aluminum or titanium reagents to both aldehydes or ketones. Starting from nucleoside and carbohydrate analogues possessing a ketone, and depending of the nature of the glycone part, the reaction can lead to diastereoselectivity.

### 2.1. Addition of Aromatic Organolithiums to Carbonyl Groups

Two strategies were developed. The first one was the direct addition of an aromatic ring to a carbonyl group starting from nucleoside analogues and the second one was the addition of an aromatic ring to the carbonyl group of carbohydrate as starting material, followed by introduction of a nucleobase.

**Scheme 1:**
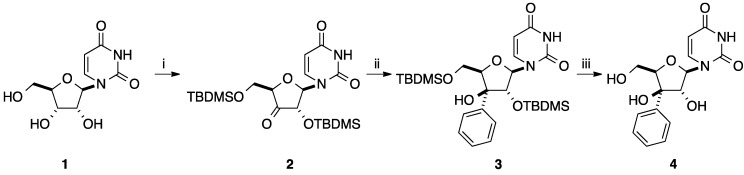
Synthesis of 3'(*S*)-*C*-phenyl-β-D-xylofuranosyluracil (**4**).

In 1987, Miyasaka and co-workers reported the synthesis of 3' (*S*)-*C*-phenyl-β-d-xylofuranosyluracil (**4**) in good yield [8]. This family of modified nucleoside analogues has been known to have potent biological activity and to be useful for elucidation of enzyme recognition of substrates. Starting from 2',5'-bis-*O*-*tert*-butyldimethylsilyl-3'-ketouridine (**2**) obtained in two steps from uridine (**1**) [9], treatment with an excess of phenyllithium in THF for 3 h at below −70 °C furnished the corresponding alcohol **3** in 72% yield. Then, classical deprotection of **3** in presence of TBAF in THF gave the corresponding triol **4** (Scheme 1). The authors did not report the presence of a diastereoisomeric mixture during the addition of the aromatic ring to the carbonyl group. Application of this approach to the synthesis of the corresponding 2'-*C*- phenyl analogue did not afford the target aromatic derivative, probably due to the known instability of 2'-ketouridine.

*Reagents and Conditions*: (i) Reference [9]; (ii) PhLi, THF, −70 °C, 3 h, 72%; (iii) TBAF, THF.

**Scheme 2:**
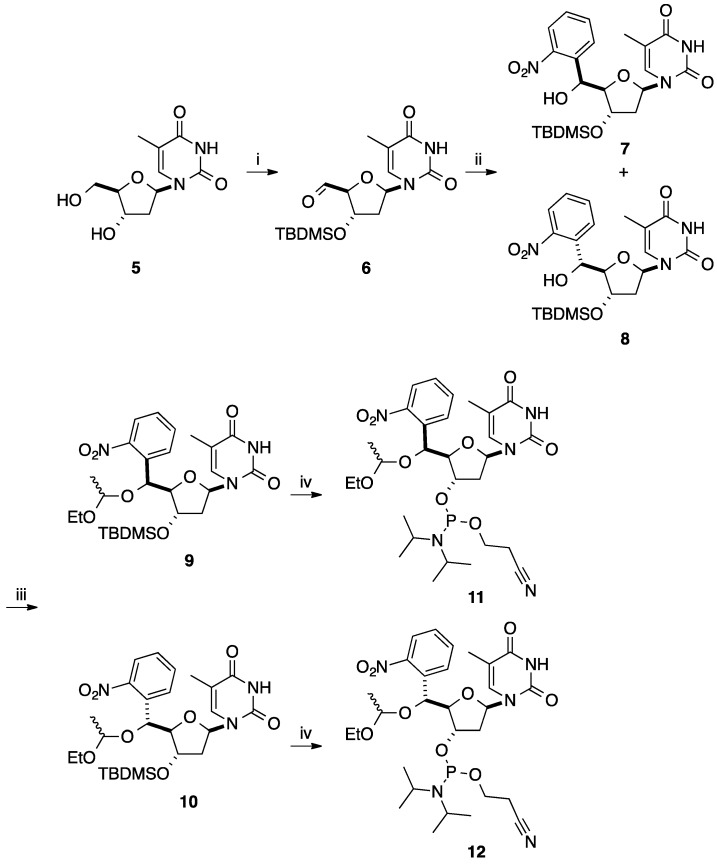
Synthesis of 5'(*S*)- and 5'(*R*)-*C*-phenyluridine analogues **11** and **12**.

A similar sequence was applied to the aldehyde **6** [10] which was obtained in four steps from thymidine (**5**) via subsequent protection of the 5'-OH group, silylation of the 3'-OH group, removal of the protection of the 5'-OH group and then Moffatt oxidation of the 5'-OH group. This strategy furnished the 5'-*C*-aryl derivatives **11** and **12** [11] as nucleotide analogues for a study on site-specific DNA cleavage [11,12]. Starting from 1-bromo-2-nitrobenzene in the presence of phenyl lithium, a metal-halide exchange in THF at −105 °C permitted obtaining an epimeric mixture of alcohols **7** and **8** (**7** (5'*S*)/**8** (5'*R*) (4.6:1) in 66% yield. The diastereoisomeric excess (de 64%) was not explained by the authors. Then, conversion of the mixture of isomers **7** and **8** gave, after flash column chromatography, the acetals **9** and **10** in 76% and 16% yields, respectively. A conventional deprotection step followed by transformation of the hydroxyl group in position 3' to a phosphoramidite afforded the intermediates **11** and **12** in 77% and 71% yields (over two steps), respectively (Scheme 2). The phosphoramidites **11** and **12** were incorporated into oligonucleotides by standard automated DNA synthesis.

*Reagents and Conditions*: (i) Reference [10]; (ii) 1-bromo-2-nitrobenzene, PhLi, THF, −105 °C, 5 h, 66%; (iii) ethylvinyl ether, PPTS, CH_2_Cl_2_, 18 h, **9**: 76% and **10**: 16%; (iv) (a) TBAF, THF, 2 h; (b) 2-cyanoethyl-*N*,*N*-diisopropylphosphorochloroamidite, (iPr)_2_EtN, CH_2_Cl_2_, 2 h, **11**: 77%, **12**: 71% for the two steps.

**Scheme 3:**
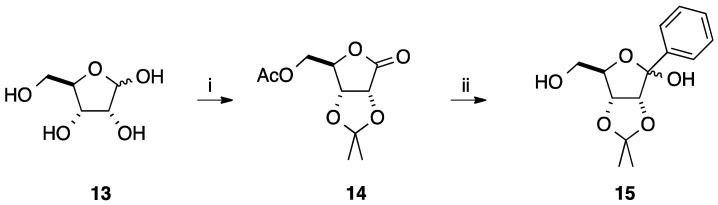
Synthesis of 1-*C*-phenyl-D-ribofuranosyl analogues **15**.

In parallel, addition of aromatic ring on a carbonyl group was realized on carbohydrate starting materials. In 2001, Sasaki and co-workers reported for the first time the synthesis of W-shape nucleic acid (WNA) designed for selective formation of anti-parallel triplexes formation [13]. WNAs are bicyclic nucleoside analogues bearing an aromatic moiety for stacking and a heterocyclic part as purine base for Hoogesteen hydrogen bonds. The strategy started from D-ribono-1,4-lactone **14** which was prepared in four steps from D-ribose (**13**) via protection of the 2,3-dihydroxy groups, acetylation of the residual hydroxyl groups, selective deacetylation and then oxidation of the anomeric position. Addition of phenyllithium in THF furnished the two anomers **15** in 53% yield [13] (Scheme 3). In the next steps, this sequence demanded protection of the primary hydroxyl group with a silyl group.

*Reagents and Conditions*: (i) (a) Acetone, H^+^; (b) Ac_2_O, pyridine; (c) piperidine, THF, 55% for the three steps; (d) PCC, CH_2_Cl_2_; (ii) PhLi, THF, −70 °C, 3 h, 53%.

**Scheme 4:**
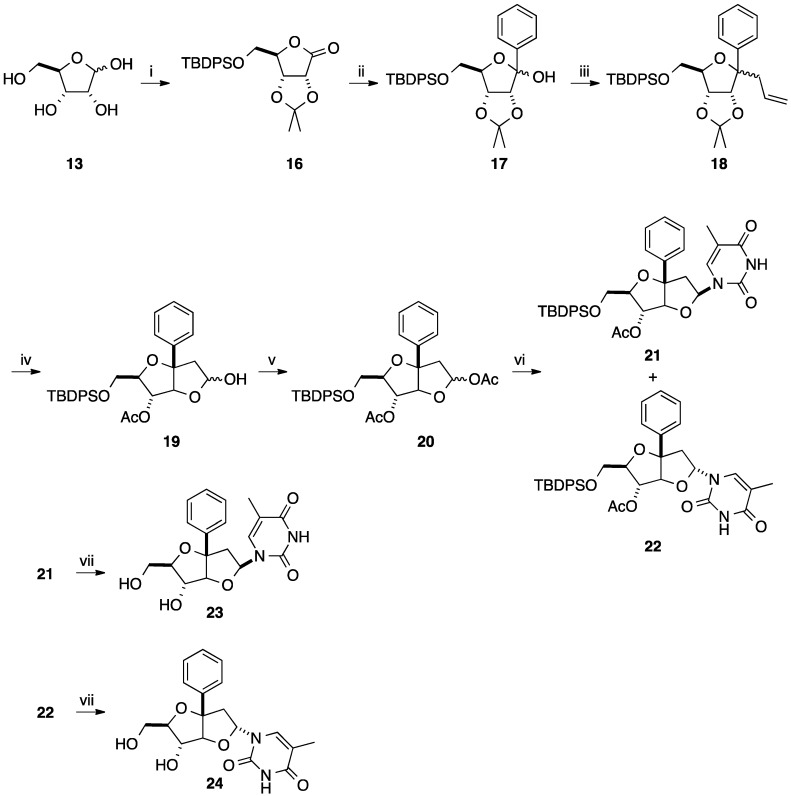
Synthesis of thymidine analogues **23** and **24**.

To complete this work, Sasaki and co-workers reported three years later a similar strategy by changing the protecting group in position 5 (silyl *vs.* acetyl) (Scheme 4) [13,14]. In this case, the *C*-*C* coupling between phenyllithium and the lactone **16** gave the two 1-*C*-phenyl lactol epimers **17** in 79% yield [14]. Allylation at the 1-position of compounds **17** gave a mixture of two anomers **18** (ratio of α/β 7:6) in 82% yield. An elegant chemical sequence for the bicyclo[3.3.0]octane derivative was reported by Sasaki and co-workers. Subsequent oxidative cleavage of the vinyl group of **18** gave the corresponding aldehyde and deprotection of the diol in position 2,3 spontaneously provided the two corresponding bicyclo[3.3.0]octane derivatives **19** in 28% yield (two steps). After acetylation of the two hydroxyl groups furnishing the two epimers **20** in 90% yield, conventional *N*-glycosidation with thymine was done to produce the target α- and β-isomers **22** and **21** in 37% and 42% yields, respectively. After flash column chromatography, each nucleoside analogues **21** and **22** were deprotected to give the corresponding diols **23** and **24** in 71% and 47% yields, respectively. After classical protection and activation steps, the corresponding phosphoramidites were incorporated to oligonucleotides by standard automated DNA synthesis.

**Figure 1:**
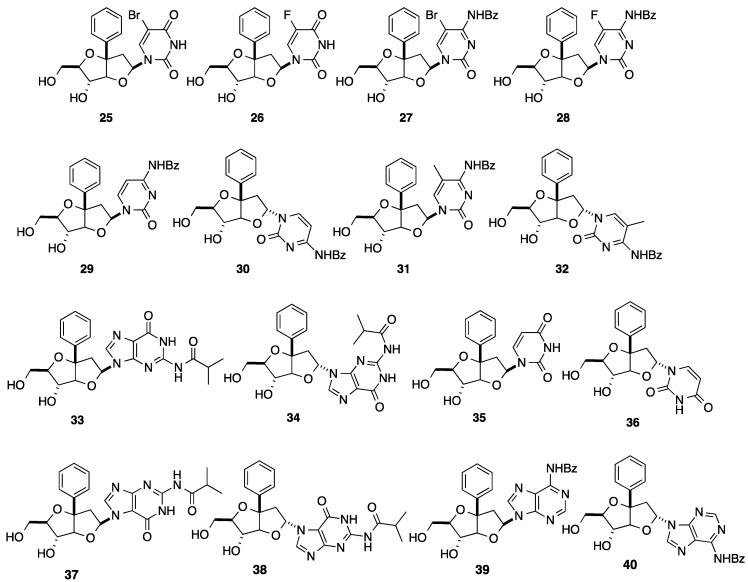
Bicyclo[3.3.0]octane nucleoside analogues **25**–**40** having a phenyl group [13,14,15].

**Figure 2:**
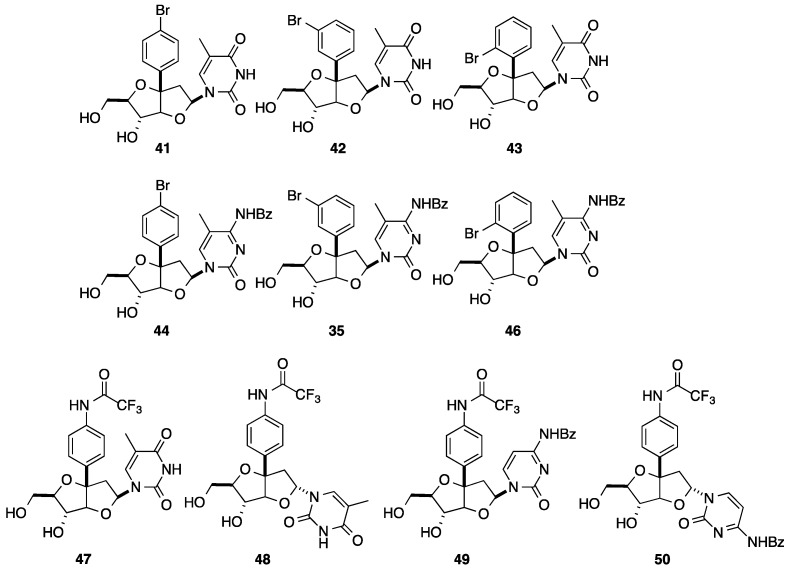
Bicyclo[3.3.0]octane nucleoside analogues **41**–**50** having a substituted aromatic ring.

At this stage, from the mixture of the key glycosyl donors **20**, the strategy described provides straightforward access in an efficient fashion to the different nucleoside analogues **25**–**40** in a bicyclo[3.3.0]octane series as presented in Figure 1 [13,14,15]. As usual, *N*-glycosidation with a guanine derivative afforded a mixture of 7-*N* and 9-*N* alkylated isomers and α- and β-isomers **33**, **34**, **37** and **38**. It is noteworthy that introduction of the nucleobase furnished in each case a mixture of two isomers, but the authors did not mention at any time the ratio of the α-isomer. In addition to the above-mentioned syntheses, Sasaki and co-workers reported the preparation of the halogeno- and amino-functionalized bicyclonucleoside analogues **41**–**50** (Figure 2) [16].

During this period, Sasaki and co-workers reported the synthesis of compounds **53** and **54** [17] using the same strategy described above [14]. In this case, acetylation of the hydroxyl group of **17** as pre-treatment for the *N*-glycosidation did not furnish the corresponding acetate but caused carbohydrate ring opening to yield the corresponding undesired acyclic derivative [18].

*Reagents and Conditions*: (i) (a) Acetone, H^+^; (b) TBDPSCl, TEA, DMAP, CH_2_Cl_2_; (c) PCC, CH_2_Cl_2_, 77% for the three steps; (ii) PhLi, THF, −78 °C, 2 h, 79%; (iii) CH_2_=CHCH_2_TMS, ZnBr_2_, CH_3_NO_2_, 0 °C then rt, 2 h, 82%; (iv) (a) aq. OsO_4_, NaIO_4_, pyridine, rt, 30 h; (b) H_2_SO_4_ (5%), THF, 60 °C, 6 h, 28% for the two steps; (v) Ac_2_O, pyridine, 0 °C, 39 h, 90%; (vi) HDMS, TMSCl, SnCl_4_, thymine, CH_3_CN, 50 °C, 4 h, **21**: 42%; **22**: 37%; (vii) (a) TBAF, THF, rt, 2 h; (b) NaOH, THF, MeOH, 0 °C, 1 h, **23**: 71%; **24**: 47% for the two steps.

**Scheme 5:**
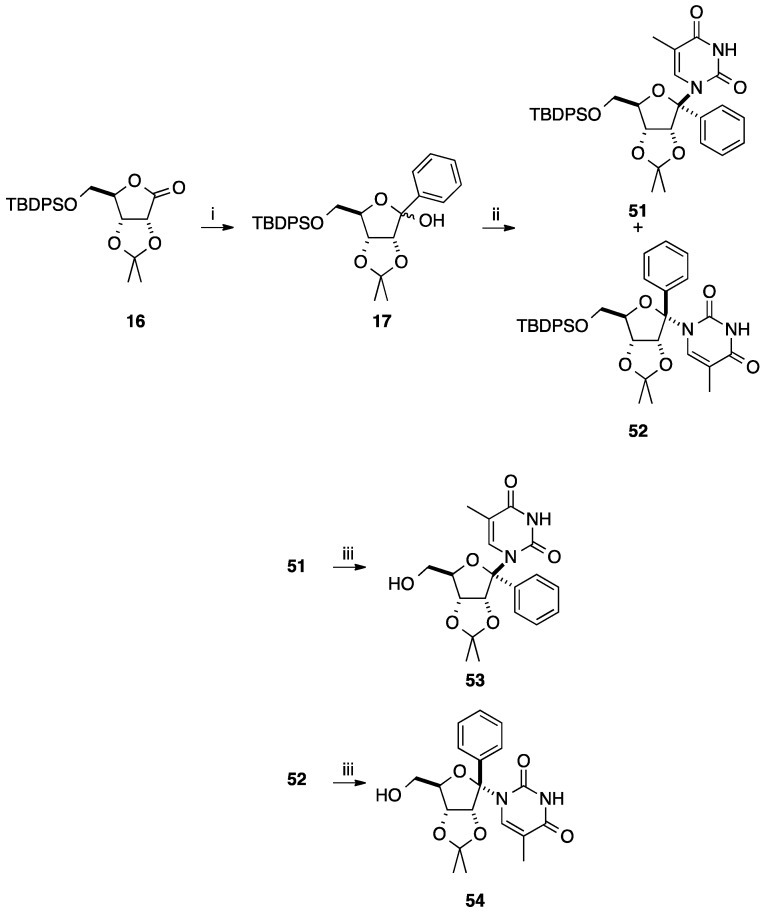
Synthesis of 1'(*R*)- and 1'(*S*)-*C*-phenyl-D-ribofuranosylthymine analogues **53** and **54**.

Due to this reactivity, the authors developed the direct *N*-glycosidation of the two epimeric alcohols **17**. Thymine was mixed in presence of the silylating agent BSA and Lewis acid TMSOTf with the epimeric mixture of **17** at 0 °C to produce the β-nucleoside **51** (α-phenyl) in 31% yield. The same reaction at 50 °C furnished a mixture of two isomers (α-nucleoside/β-nucleoside, **52**/**51**, 6:31) showing that the β-nucleoside **51** was formed by thermodynamic process. Then, classical deprotection of the primary hydroxyl group of **51** and **52** afforded the nucleoside analogues **53** and **54** in 49% and 63% yields, respectively (Scheme 5).

*Reagents and Conditions*: (i) PhLi, THF, −78 °C then rt, 4 h, 71%; (ii) thymine, BSA, TMSOTf, 50 °C, 4.5 h, **51**: 31%, **52**: 6%; (iii) TBAF, THF, rt, 2 h, **53**: 49%, **54**: 63%.

**Figure 3:**
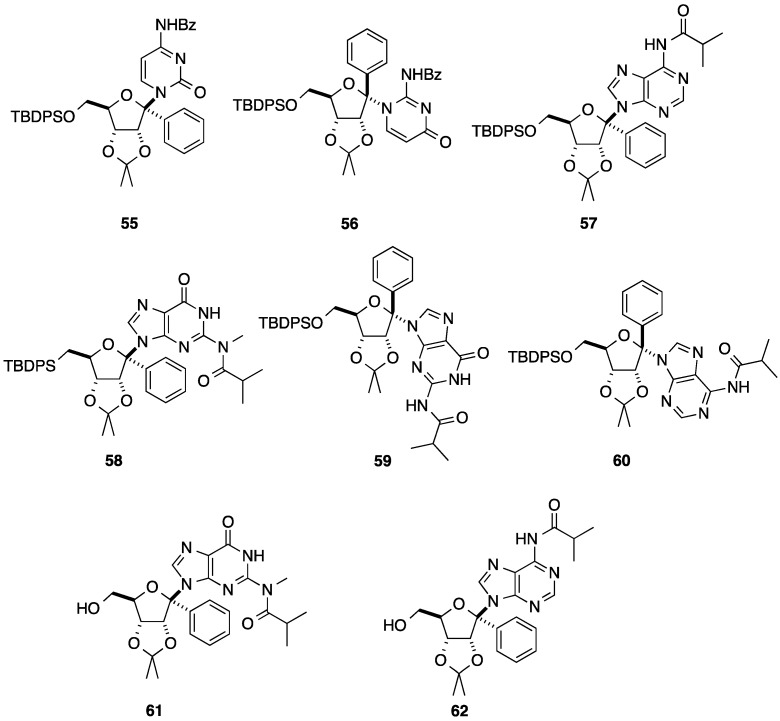
1'(*R*)- and 1'(*S*)-*C*-phenyl-D-ribofuranosylnucleoside analogues **55**–**62**.

Introduction of all four nucleobases were realized using similar strategy giving compounds **55**–**60** and two of them were selectively deprotected to obtain the β-isomers **61** and **62** (Figure 3). In 1982, Vasella and co-workers reported the synthesis of 4'-*C*-aryl-D-ribonucleosides as synthons for the synthesis of antibiotics [19]. Starting from the 1,4-lactone derivative **64** obtained from the ribonolactone **63** in two steps, addition of an excess of 2-methoxymethoxyphenyllithium at 10 °C afforded two isomeric lactones **65** and **66** in 65% yield with an excess of the L-lyxo **66** (54%). In order to have more D-ribose derivative, the authors described the conversion of the L-lyxo form **66** to the target D-ribo form **65** in 89% yield by treatment with piperidine and the addition of methanesulfonyl chloride and TEA. Reduction of the isolated lactone **65** with DiBAL-H afforded the two lactols **67** in 95% yield and then subsequent deprotection of the diol and acetylation of the free hydroxyl group gave the glycone derivatives **68** in 90% yield, respectively.

**Scheme 6:**
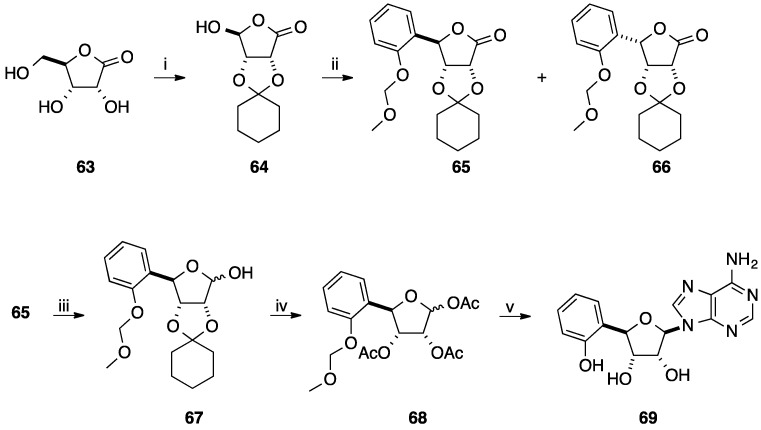
Synthesis of (4'(*R*)-*C*-phenyl-D-ribo-tetrofuranosyl)adenine analogue **69**.

*Reagents and Conditions*: (i) (a) Cyclohexanone, FeCl_3_, Sikkon, 50 °C, 2.5 h, 89%; (b) NaOH, H_2_O, NaIO_4_, 0 °C then BaCl_2_.10 H_2_O, 4 °C, 10 min, 93%; (ii) CH_3_OCH_2_OPhLi, Et_2_O, 10 °C, 3 h, **65**: 30%, **66**: 35%; (iii) DIBAL-H, toluene −78 °C, 10 min, 95%; (iv) (a) aq. HCl (0.2 M), 50 °C, 7 h; (b) Ac_2_O, pyridine, 90% for the two steps; (v) (a) *N*^6^-benzoyladenine, HDMS, TMSOTf, CH_3_CN, 60 °C, 1 h; (b) NH_3_, MeOH, rt, 48 h, 68% for the two steps.

Using the Vorbrüggen methodology, addition of *N*6-benzoyladenine to the mixture of anomers **68** in presence of TMSOTf and HDMS afforded selectively the β-isomer via a C2 acetyloxonium intermediate. Then, direct treatment of the nucleoside analogue with NH_3_ in methanol led to the target adenosine derivative **69** in 68% yield (two steps) (Scheme 6).

### 2.2. Addition of Aromatic Organomagnesium Reagents to Carbonyl Groups or Analogues

Using aromatic organomagnesium reagents, two strategies were developed starting from either a nucleoside analogue or from a carbohydrate derivative. Substitution of phenyllithium by the corresponding Grignard reagent was described by Miyasaka and co-workers for the synthesis of 3'(*S*)-*C*-phenyl-β-D-xylofuranosyluracil (**4**). Unfortunately the target compound was obtained in poor yield (30%) (see Scheme 1) [8].

**Scheme 7:**
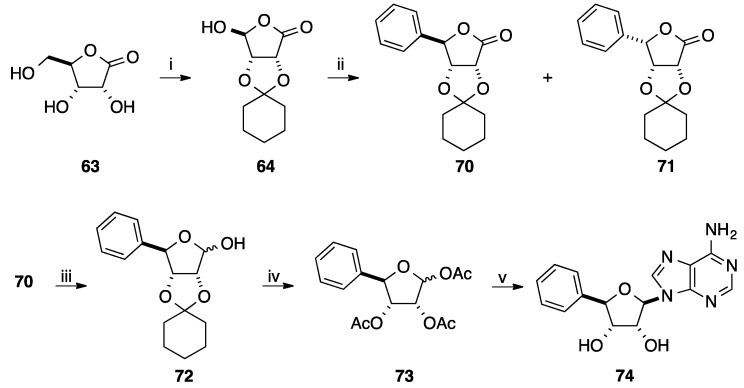
Synthesis of (4'(*R*)-*C*-phenyl-D-ribotetrofuranosyl)adenine analogue **74**.

Vasella and co-workers have also reported in the same paper described above the use of phenylmagnesium bromide instead 2-methoxymethoxyphenyllithium for the synthesis of 4'-*C*-aryl-D-ribonucleoside analogue [19]. Starting from the platform molecule **64**, addition of an excess of phenylmagnesium bromide at 10 °C afforded two isomeric lactones **70** and **71** in 81% yield. Attempts to improve the diastereoselectivity of the Grignard reaction showed that the ratio varied between 58:42 (10 °C, normal addition) and 25:75 (−40 °C, inverse addition). Then following the same strategy, reduction of the lactone **70**, deprotection and acetylation, *N*-glycosidation and treatment in basic media conducted to the target adenosine derivative **74** in 58% yield (four steps) (Scheme 7).

*Reagents and Conditions*: (i) (a) Cyclohexanone, FeCl_3_, Sikkon, 50 °C, 2.5 h, 89%; (b) NaOH, H_2_O, NaIO_4_, 0 °C then BaCl_2_.10 H_2_O, 4 °C, 10 min, 93%; (ii) PhMgBr, THF, 10 °C, 2 h, **70**: 47%, **71**: 34%; (iii) DIBAL-H, toluene, −78 °C, 10 min, 99%; (iv) aq. AcOH, 60 °C, 2 h, 89%; (v) (a) *N*^6^-benzoyl-*N*^6,9^-bis(trimethylsilyl)adenine, SnCl_4_, CH_2_Cl_2_, rt, 1 h; (b) NH_3_, MeOH, rt, 15 h, 66%.

**Scheme 8:**
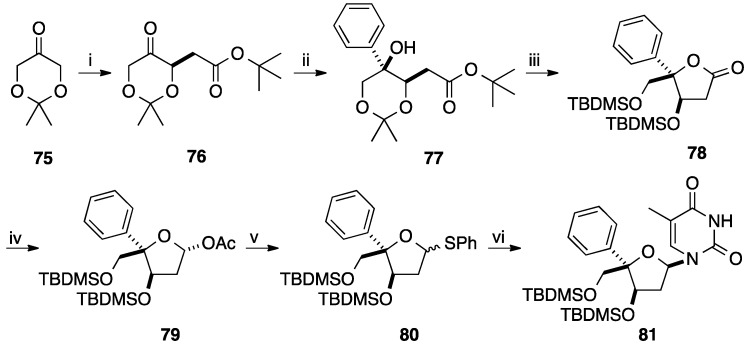
Synthesis of 5(*R*)-*C*-phenyltetrahydrofurane derivative **81**.

In 2008, Enders and co-workers developed an elegant strategy for the preparation of 4'-*C*-arylnucleosides [20]. A versatile and efficient route for the selective synthesis of the platform molecule **78** having two asymmetric carbon atoms was described. Starting from the achiral 2,2-dimethyl-1,3-dioxan-5-one (**75**), α-alkylation using RAMP-hydrazone methodology furnished enantioselectively the corresponding ester **76** in 57% yield (three steps) [21]. Diastereoselective Grignard reaction afforded, after flash chromatography, the major *syn* diastereoisomer **77** in 88% yield (Scheme 8).

*Reagents and Conditions*: (i) Reference [21]; (ii) PhMgBr, THF, −78 °C then flash chromatography, 88%; (iii) (a) HCl (3N), MeOH, rt; (b) TBDMSOTf, pyridine, THF, 0 °C, 89% for the two steps; (iv) (a) DIBAL-H, CH_2_Cl_2_, −78 °C; (b) Ac_2_O, pyridine, rt, 62% for the two steps; (v) TMSSPh, BF_3_.OEt_2_, hexane, −95 °C to rt, 89%; (vi) Bis-TMS-thymine, NBS, 4-A molecular sieve, CH_2_Cl_2_, −78 °C to −26 °C, 87%.

**Figure 4:**
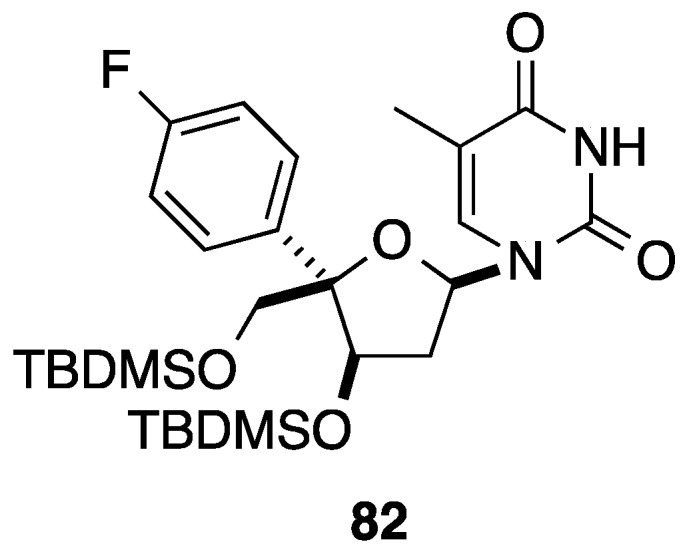
5(*R*)-(4-Fluorophenyl)tetrahydrofuran derivative **82**.

Conventional cleavage of the acetonide, subsequent cyclization giving the lactone and then protection of the residual two hydroxyl groups afforded the corresponding 5-phenyltetrahydrofuran analogue **78** in 89% yield (two steps). Reduction of the lactone **78** with DIBAL-H and subsequent acetylation of the lactol furnished selectively the acetal **79** in 62% yield. The authors reported that only the α-anomer was observed. Instead of directly using the acetal **79**, Enders and co-workers preferred to convert compound **79** to the corresponding mixture of thioglycosides **80** in 89% yield. Then, a classical silyl-Hilbert-Johnson reaction was applied to give the thermodynamically more stable β-anomers **81** in 87% yield. No attempt to remove the protecting group on compound **81** was mentioned. Application of this strategy furnished the fluoro derivative **82** (Figure 4).

### 2.3. Addition of Aromatic Organoaluminium Reagents to Carbonyl Groups

**Scheme 9:**
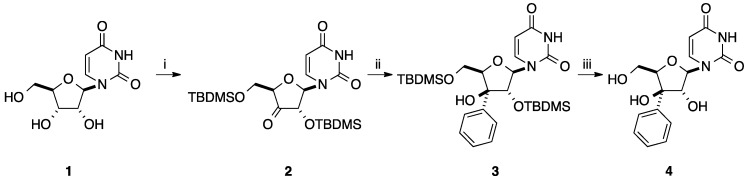
Synthesis of 3'(*S*)-*C*-phenyl-β-D-xylofuranosyluracil (**4**).

Addition of aromatic organoaluminium reagents was described starting from either nucleoside analogues or carbohydrate derivatives. Substitution of phenyllithium by the corresponding phenylaluminium reagent was described by Miyasaka and co-workers for the synthesis of 3'-*C*-phenyluridine analogue **4** (Scheme 9) [8]. The carbalumination of the ketone **2** was attempted in the presence of an excess of phenylaluminium in CH_2_Cl_2_ at −70 °C, but no reaction occurred. It needed to run at room temperature for one hour. The authors reported that even under reflux no decomposition was observed. This reaction led selectively to the nucleoside analogue **3** which could not be isolated in pure form. Subsequent deprotection of the alcohol **3** gave the target nucleoside analogue **4** in 26% yield (two steps). This sequence using Ph_3_Al permitted to prepare compound **4**, but with a lower yield than that using PhLi.

*Reagents and Conditions*: (i) Reference [9]; (ii) Ph_3_Al, CH_2_Cl_2_, rt, 1 h; (iii) TBAF, THF, rt, 2 h, 26% for the two steps.

**Scheme 10:**
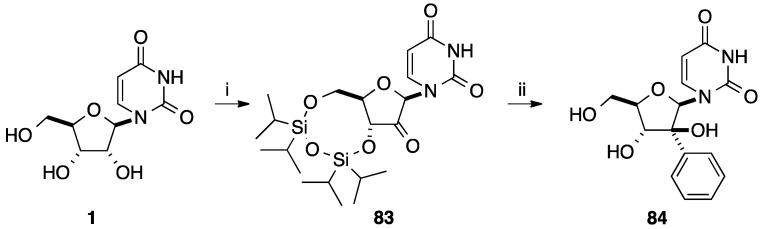
Synthesis of 2'(*S*)-*C*-phenyl-β-D-arabinofuranosyluracil (**84**).

Application of this method from the 2'-keto derivative **83** [22] was realized to furnish the 2'(*S*)-*C*-phenyluridine analogue **84** in 30% yield (Scheme 10).

*Reagents and Conditions*: (i) Reference [22]; (ii) (a) Ph_3_Al, CH_2_Cl_2_, rt, 1 h; (b) TBAF, THF, rt, 2 h, 30% for the two steps.

### 2.4. Addition of Aromatic Organotitanium Reagents to Carbonyl Groups or Analogues

**Scheme 11:**
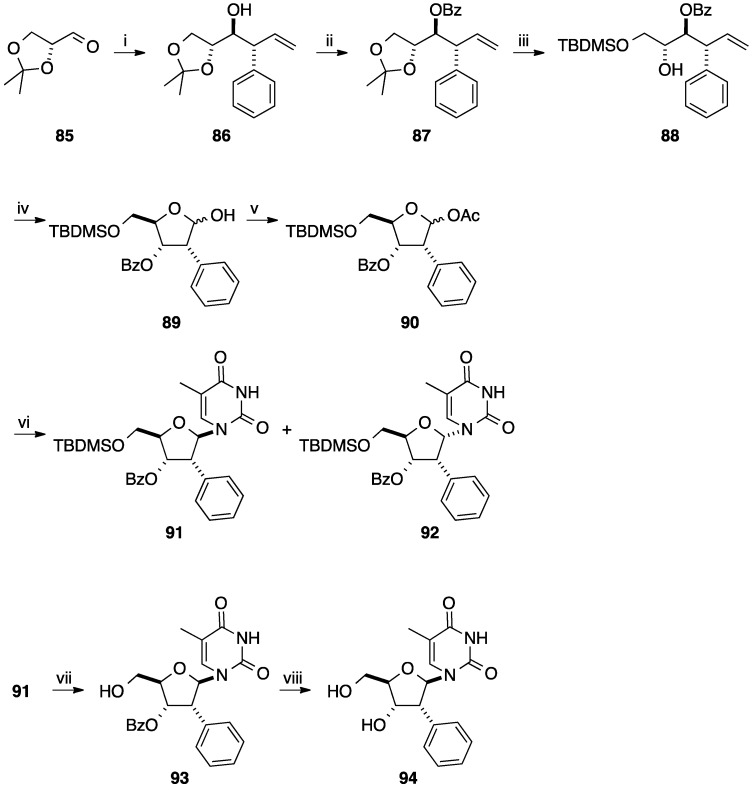
Synthesis of (2(*R*)-*C*-phenylribofuranosyl)thymine derivative **94**.

Using chiral titanium complexes, addition to aldehydes led enantioselectively to the corresponding alcohol as a platform for the synthesis of 2(*R*)-*C*-phenyl carbohydrate derivatives. In 1992, Duthaler and co-workers reported the synthesis of 2-*C*-phenylribofuranosyl analogues [23]. Starting from aldehyde **85**, an (*R*,*R*)-configured allyltitanium reagent was added to glyceraldehyde **85** to furnish the corresponding allyl derivative **86** in 75% yield. The diastereoselectivity was excellent and exclusive *Si*-face addition was observed. After successive benzoylation of the secondary hydroxyl group of **86**, deprotection of the diol and silylation of the primary hydroxyl group gave the allylic compound **88**. Ozonolysis of the vinyl bond of **88** afforded the resulting lactol **89** in 50% (four steps) [24,25]. Acetylation of the glycone **89** and *N*-glycosidation using the Vorbrüggen methodology furnished a mixture of the two anomers **91** and **92** (**91**–**92**, 3.5/1). After flash chromatography and classical deprotection of the primary hydroxyl group the target nucleoside analogue **93** was obtained (Scheme 11). Modification of compound **94** permitted its incorporation into oligonucleotides by standard automated DNA synthesis.

*Reagents and Conditions*: (i) Reference [23]; (ii) BzCl, pyridine; (iii) (a) TFA, MeOH; (b) TBDMSOTf, TEA, CH_2_Cl_2_; (iv) O_3_, MeOH, −78 °C and then Me_2_S, −78 °C, rt, 50% for the five steps; (v) Ac_2_O, DMAP, pyridine, CH_2_Cl_2_, 0 °C-rt, 87%; (vi) (a) thymine, BSA C_2_H_4_Cl_2_, 83 °C; (b) TMSOTf, C_2_H_4_Cl_2_, rt, **91**: 53% **92**: 15%;(vii) TBAF, THF, rt; (viii) MeONa, MeOH, rt.

**Figure 5:**
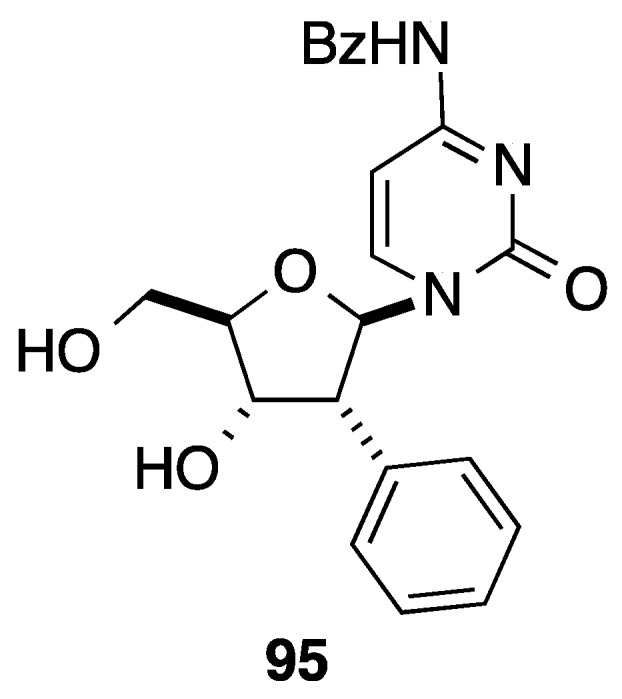
(2(*R*)-*C*-Phenylribofuranosyl)cytosine derivative **95**.

In this paper, the authors used the previous intermediates **90** to prepare the cytosine analogue **95** following a classical methodology of nucleobase insertion and hydroxyl deprotection (Figure 5).

## 3. Cross-Coupling Reactions

Palladium-catalyzed cross coupling reactions were studied to obtain mainly d4T analogues having 2',3'-didehydro-2',3'-dideoxy-D-ribose as the glycone part. Two main strategies were developed: the first one was the formation of a bromovinyl intermediate or analogue and the second one the formation of an unsaturated stannyl intermediate.

**Scheme 12:**
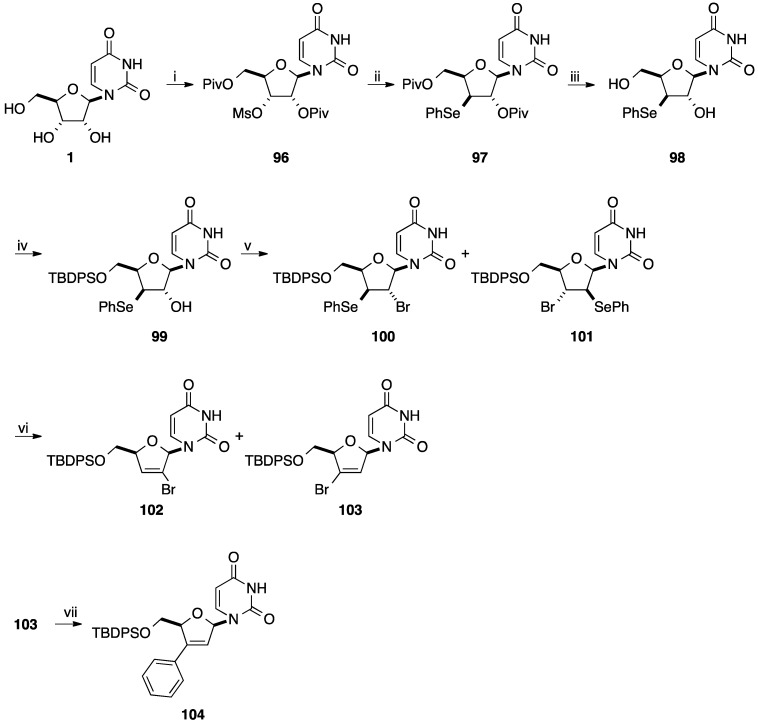
Synthesis of 3'-*C*-phenyl d4U analogue **104**.

Tanaka and co-workers described the synthesis of 2'-*C*- and 3'-*C*-branched 2',3'-unsaturated nucleosides via palladium-catalysed cross-coupling of the bromovinyl intermediates [26,27]. The 3'-bromo derivative **103** was prepared starting from uridine (**1**) in a multi-step sequence. Treatment of the 3'-*O*-mesyl derivative **96** [28] with (PhSe)_2_ in presence of NaBH_4_ in refluxing THF-EtOH gave selectively the phenylseleno derivative **97** in 81% yield with inversion of configuration at the 3' position. After perdeacylation and selective silylation of the primary hydroxyl group, bromination in the presence of SOBr_2_ and imidazole in CCl_4_ afforded a mixture of β-bromoselenides **100** and **101**. Then without purification, the crude mixture of the regioisomers **100** and **101** was submitted to a selenoxide elimination. Treatment of **100** and **101** with MCPBA in CH_2_Cl_2_ furnished the bromovinyl derivatives **102** and **103** in 38% and 42% yields, respectively. After flash chromatography, compound **103** was subjected to a Stille reaction using organotin reagents, as coupling partners, in presence of (Ph_3_P)_2_PdCl_2_ (10 mol %) in dioxane at 100 °C for 24 h to obtain the nucleoside analogue **104** in 39% yield (Scheme 12). The same group reported two years later that application to the Stille reaction starting from the bromovinyladenine nucleoside analogue did not give any phenyl derivative [27].

**Scheme 13:**
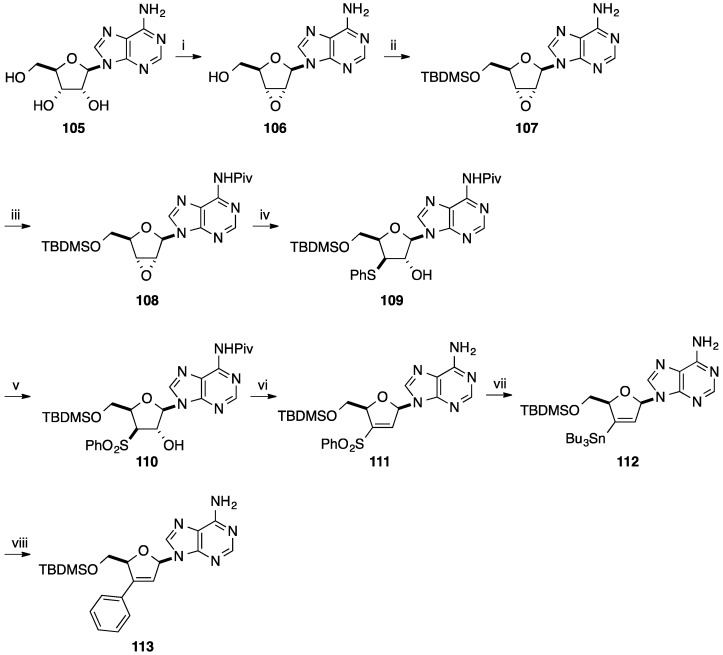
Synthesis of 3'-*C*-phenyl d4A analogue **113**.

Tanaka and co-workers described the synthesis of 3'-*C*-phenyl-d4A analogue **113** by radical-mediated desulfonylative stannylation [29]. Starting from the epoxide **106** obtained from adenine (**105**) [30], silylation of the hydroxymethyl group by a conventional method gave the epoxide **107** in 88% yield. To avoid the oxidation of the amino group of adenine, pivaloylation of **107** gave the protected adenine derivative **108** in 97%. Then, selective ring opening by addition of thiophenolate gave the thioether **109** in 90%. Compound **109** was submitted to MCPBA oxidation to give the β-hydroxysulfone product **110** in quantitative yield. Deprotection of the amino group of compound **100** and subsequent methylsulfonylation directly afforded the *cis*-elimination product **111** in 81% yield (two steps). Radical-mediated desulfonylative stannylation of **111** proceeded efficiently by reacting with Bu_3_SnH in the presence of AIBN and triethylamine in refluxing benzene to give the 3'-*C*-stannyl nucleoside **112** in 76% yield (Scheme 13). With the 3'-*C*-stannyl derivative **112** in hands, the 3'-*C*-phenyl analogue **113** was prepared by the Stille reaction in presence of PhI, Pd(PPh_3_)_4_ and CuI in DMF at room temperature for 28 h. The target aromatic derivative **113** was obtained in 66% yield.

*Reagents and Conditions*: (i) Reference [28]; (ii) (PhSe)_2_, EtOH, NaBH_4_, THF, reflux, 48 h, 81%; (iii) aq NaOH (1N), EtOH, rt, 24 h; (iv) TBDMSCl, pyridine, rt, 24 h, 96% for the two steps; (v) imidazole, SOBr_2_, CCl_4_, rt, 6 h; (vi) MCPBA, rt, 12 h, **102**: 38%, **103**: 42% for the two steps; (vii) Ph_4_Sn, (Ph_3_P)_2_PdCl_2_ (10 mol %), TEA, DMF, 100 °C, 24 h, 39%.

**Scheme 14:**
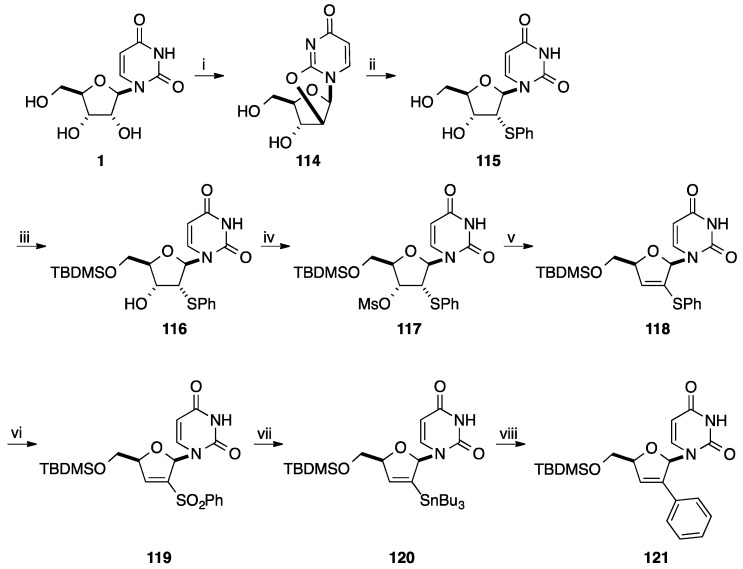
Synthesis of 2'-*C*-phenyl d4U analogue **121**.

The same year, Tanaka and co-workers developed a similar strategy for the preparation of 2'-*C*-phenyl d4U analogue **121** starting from uridine (**1**) [31] (Scheme 14). The main difference was the oxidation step of the phenylthio group to the benzenesulfonyl group which was realized at the end of the strategy (Scheme 14). Starting from uridine (**1**), the *O*^2^,2'-anhydrouridine **114** furnished selectively the 2'-phenylthio derivative **115** [32]. It is clear that only the anhydro strategy led to the desired selectivity. Then, selective protection of the primary hydroxyl group followed by mesylation of the 3'-OH group and elimination afforded the vinyl derivative **118** in 80% yield (three steps). Oxidation of the phenylthio derivative **118** was realized by treatment with MCPBA in methanol and gave the benzenesulfonyl derivative **119** in 79% yield. Classical radical reaction permitted to prepare the 2'-stannyl derivative **120** in 35% yield with a recovered material **119** (40%). The Stille coupling reaction between compound **120** and PhI in presence of Pd(Ph_3_P)_4_, CuI afforded the target 2'-*C*-phenyl d4U **121** in 80% yield.

*Reagents and Conditions*: (i) Reference [30]; (ii) TBDMSCl, imidazole, DMF, 0 °C, 25 min, 88%; (iii) PivCl, iPr_2_NEt, CH_2_Cl_2_, 0 °C, 1.5 h, 97%; (iv) PhSH, NaOH, MeOH, reflux, 2.5 h, 90%; (v) MCBPA, MeOH, 0 °C, 2.5 h, 100%; (vi) (a) NH_3_, MeOH, 4 °C, 12 h; (b) MsCl, DMAP, pyridine, 0 °C then rt, 12 h, 81% for the two steps; (vii) Bu_3_SnH, AIBN, TEA, benzene, 80 °C, 5.5 h, 76%; (viii) PhI, Pd(PPh_3_)_4_ (10 mol %), CuI, DMF, rt, 28 h, 66%.

**Scheme 15:**
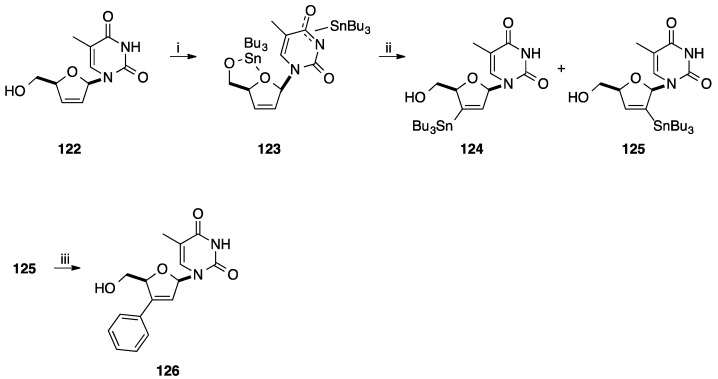
Synthesis of 3'-*C*-phenyl d4T **126**.

In order to decrease the number of step for the preparation of 2'-*C*-phenyl- and 3'-*C*-phenyl-2',3'-didehydro-2',3'-dideoxynucleoside, Tanaka and co-workers developed the direct stannylation of D4T **122** (Scheme 15) [33]. Starting from unprotected d4T **122**, stannylation was carried out using Bu_3_SnOMe at 90 °C for 90 min and furnished the bis-tributylstannyl d4T **123**. Subsequently, compound **123** was mixed with a solution of LTMP containing TMEDA at −70 °C for 15 min.

*Reagents and Conditions*: (i and ii) Reference [32]; (iii) TBDMSCl, pyridine, rt, 15 h, 100%; (iv) MsCl, pyridine, rt, 12 h, 80%; (v) DBN, CH_3_CN, reflux, 2 h, 100%; (vi) MCPBA, MeOH, rt, 3 h, 79%; (vii) Bu_3_SnH, AIBN, benzene, 80 °C, 6 h, 35%; (viii) PhI, Pd(Ph_3_P)_4_, CuI, DMF, rt, 20 h, 80%.

*Reagents and Conditions*: (i) Bu_3_SnOMe, 90 °C, 1.5 h; (ii) LTMP, TMEDA, THF, −70 °C, 15 min, **124**: 60%, **125**: 9% for the two steps; (iii) PhI, Pd(PPh_3_)_4_, CuI, DMF, rt, 12 h, 97%.

A mixture of two regioisomers were obtained, the target 3'-*C*-stannyl derivative **124** in 60% yield and the isomer 2'-*C*-phenyl derivative **125** in 9% yield. Starting from compound **124**, conventional Stille cross-coupling with PhI in presence of Pd(PPh_3_)_4_ and CuI permitted to prepare the target nucleoside analogue **126** in 97% yield.

**Figure 6:**
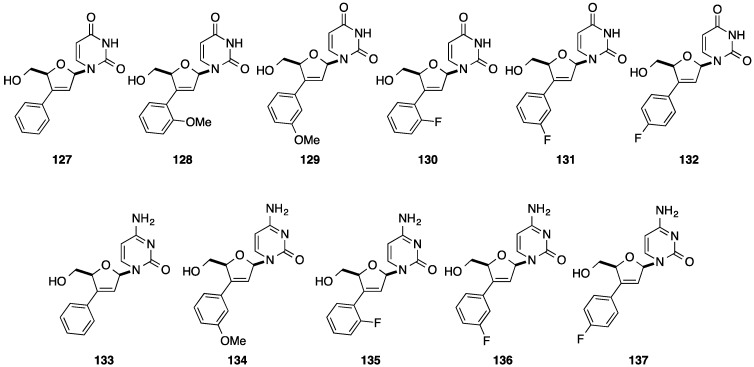
3'-*C*-Aryl d4U **127**–**132** and 3'-*C*-aryl d4C **133**–**137**.

Few years later, Tanaka and co-workers reported the same strategy starting from d4U [34]. Application of the aforementioned strategy permitted the synthesis of the d4U analogues **127**–**132**. Conversion of the uridine analogues **127** and **129**–**132** using Reese methodology furnished the corresponding 3'-*C*-aryl d4C analogues **133**–**137**, respectively (Figure 6).

**Scheme 16:**
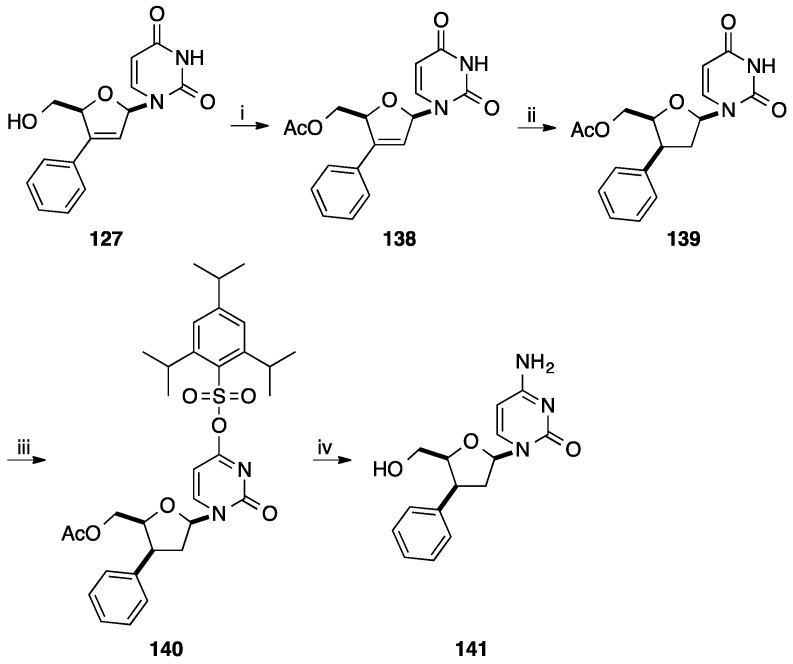
Synthesis of 3'(*S*)-*C*-phenyl ddC analogue **141**.

*Reagents and Conditions*: (i) Reference [34]; (ii) H_2_, Pd/C (5%), EtOH, EtOAc, rt, 4 days; (iii) 2,4,6-triisopropylbenzenesulfonyl chloride, DMAP, Et_3_N, 0 °C, 3 h; (iv) (a) aq NH_3_ (28%), rt, 1.5 h; (b) Ac_2_O, DMAP, iPr_2_NEt, CH_2_Cl_2_, 0 °C, 30 min; (c) NH_3_, MeOH, 5 °C, 6 days, 81% for the five steps.

**Figure 7:**
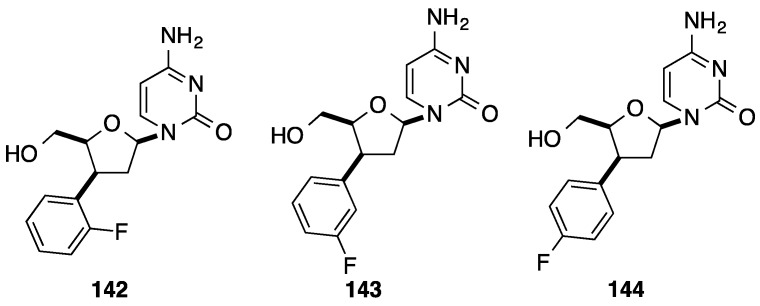
3'(*S*)-*C*-aryl ddC **142**–**144**.

Using the 3'-*C*-aryl d4C derivatives **133** and **135–137**, Tanaka and co-workers described the synthesis of the corresponding ddC analogue **141**–**144** (Scheme 16 and Figure 7) [34]. After protection of the 5'-OH group of compound **127** with an acetate, catalytic hydrogenation occured stereoselectively to give the 3'-β-phenyl analogue **139** in 88% yield then subsequent conversion of the uracile moiety to the cytosine one using Reese methodology permitted to prepare the ddC structure **141** in 88% yield (5 steps) (Scheme 16). Application of the aforementioned strategy permitted the synthesis of the ddC analogues **142**–**144** with the same diastereoselectivity (Figure 7).

## 4. Addition of Aromatic Rings to an Epoxide

**Scheme 17:**
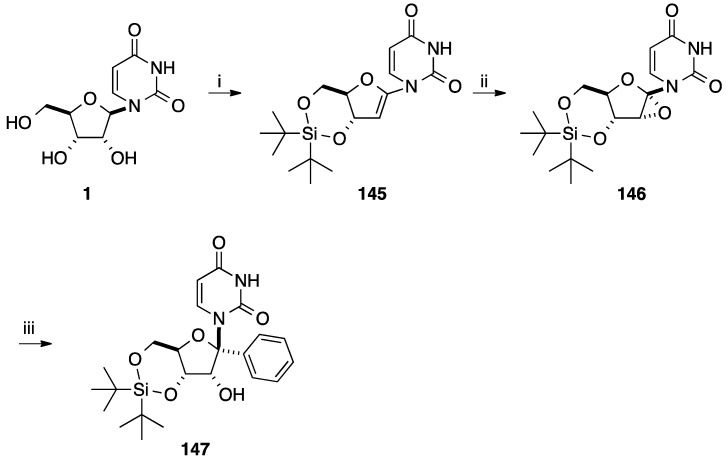
Synthesis of 1'(*R*)-*C*-phenyl uridine analogue **147**.

Introduction of an aromatic core can occur via the attack of an aromatic organoaluminium reagent to a nucleoside analogue having an epoxide. In this regards, Haraguchi and co-workers reported the ring opening of a nucleoside 1',2'-epoxide with an organoaluminium reagent for the preparation of the 1'-*C*-phenyl uridine analogue **147** [35]. The strategy developed by the authors was to start with the 1',2'-unsaturated nucleoside analogue **145** obtained in three steps from uridine (**1**) (Scheme 17) [36].

*Reagents and Conditions*: (i) Reference [36]; (ii) dimethyldioxirane, acetone, CH_2_Cl_2_; (iii) Ph_3_Al, CH_2_Cl_2_, −30 °C, 4.5 h, 55%.

Then, selective epoxidation of compound **145** was realized with an acetone solution of dimethyldioxirane and furnished only the 1',2'-α-epoxide **146**. Nucleoside analogue **146** reacted with an excess of triphenylaluminium in CH_2_Cl_2_ at −30 °C for 4.5 h. In this case, preferential formation of the *syn*-ring-opened β-anomer **146** was seen giving only the α-phenyl derivative **147** in 55% yield.

**Scheme 18:**
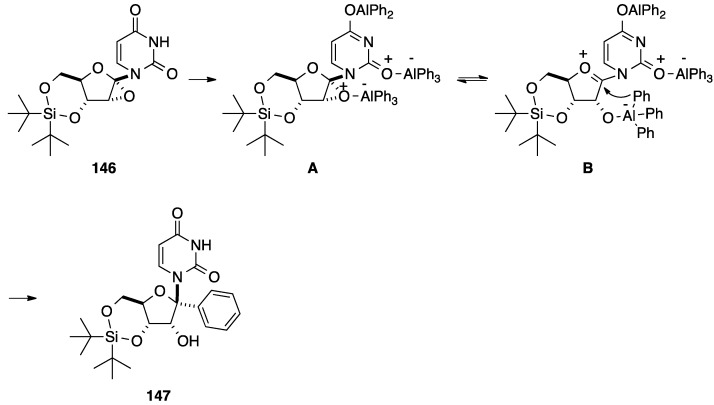
Possible reaction pathway for the synthesis of 1'(*R*)-*C*-phenyl uridine analogue **147**.

The authors proposed a possible reaction pathway for this reaction (Scheme 18). With an excess of organoaluminium reagent, the epoxide **146** gave the trialuminium derivative **A** which formed the oxonium intermediate **B**. Finally the epoxide acted as a directing group in the presence of the triphenylaluminium reagent, then a nucleophilic attack of the phenyl ligand occurred on the α face of the glycone part and furnished only the *syn*-ring-opened product **147** [35].

## 5. Cyclization

The formation of a highly functionalized aromatic core via catalytic [2+2+2]-alkyne cyclotrimerization has been well described [37]. This cyclization was reported on the glycone moiety either before the *N*-glycosidation or after.

**Scheme 19:**
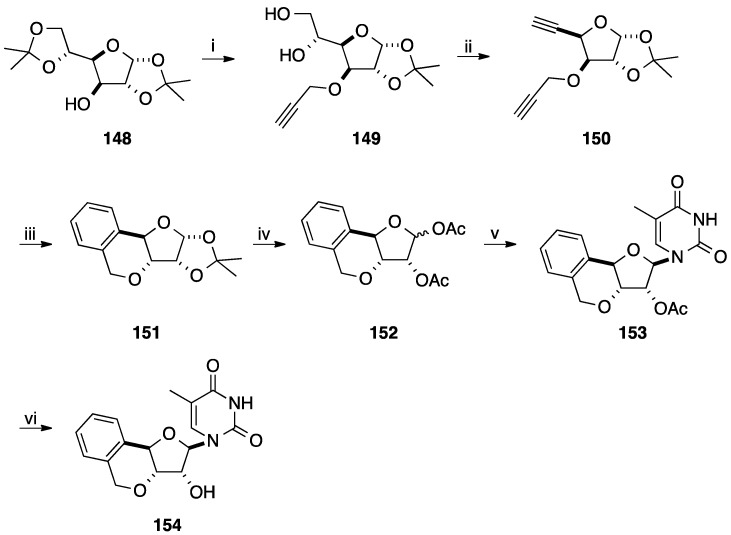
Synthesis of isochroman derivative **154**.

In order to identify new therapeutic candidates, Ramana and co-workers reported the synthesis of tricyclonucleosides having a 3-*O*,4-*C*-(*o*-phenylenemethylene) moiety using a cyclotrimerization of the sugar part and then *N*-glycosidation [38,39]. Starting from the diol **149** obtained from 1,2-5,6-di-*O*-isopropylidene-α-D-glucose (**148**) [40], sodium metaperiodate mediated cleavage and subsequent Ohira-Bestmann alkynylation of the aldehyde furnished the corresponding diyne **150** in 78% yield (two steps). Compound **150** under an acetylene atmosphere in the presence of Wilkinson's catalyst in toluene was first mixed at −78 °C during 25 min to give after 4 h at 80 °C the desired xylotetrofuranose derivative **151** in 65% yield. After deprotection and acetylation of the diol, a mixture of the two anomers **152** was obtained in 87% yield. Due to the assistance of the acetyl group in position 2' under conventional nucleobase insertion conditions, the two isochroman derivatives **152** gave selectively the protected β-nucleoside analogue **153** in 79% yield. Then, classical deprotection of the residual secondary hydroxyl group furnished the target copound **154** in 95% yield (Scheme 19).

*Reagents and Conditions*: (i) Reference [40]; (ii) (a) NaIO_4_, MeOH, rt, 30 min; (b) K_2_CO_3_, Ohira-Bestmann reagent, MeOH, rt, 6 h, 78% for the two steps; (iii) acetylene, toluene, RhCl(PPh_3_)_3_, toluene, −78 °C, 25 min then 80 °C, 4 h, 65%; (iv) (a) aq AcOH (60%), reflux, 2 h; (b) Ac_2_O, TEA, DMAP, CH_2_Cl_2_, 0 °C, 1 h then rt, 1 h, 87% for the two steps; (v) thymine, BSA, CH_3_CN, reflux, 15 min then TMSOTf, 50 °C, 2 h, 79%; (vi) MeONa, MeOH, rt, 20 min, 95%.

**Figure 8:**
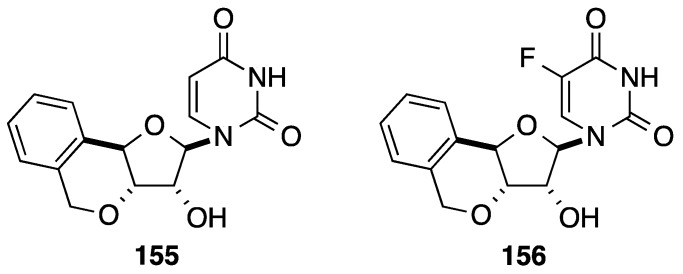
Isochroman derivatives **155** and **156**.

Application of the aforementioned procedure permitted to prepare the different analogues **155** and **156** (Figure 8).

**Scheme 20:**
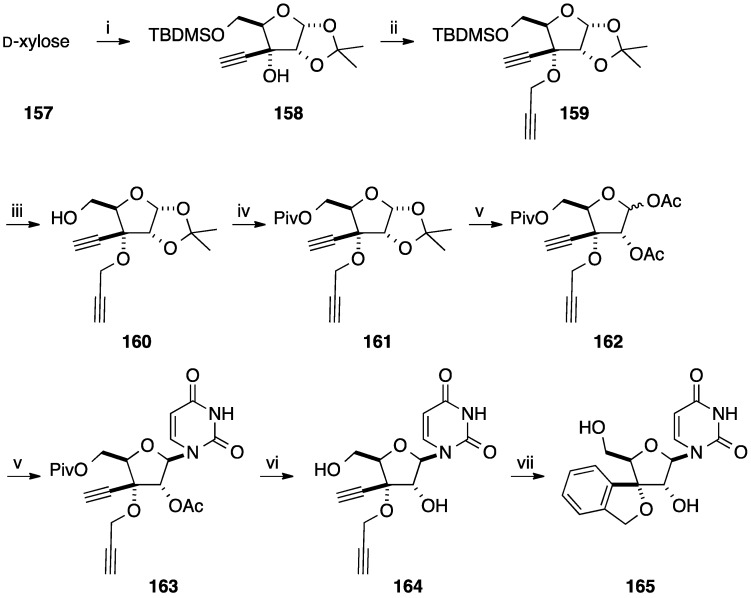
Synthesis of the 3'-*C*-spiro uridine analogue **165**.

The strategy developed by Ramana and co-workers did not permit preparation of the 3'-*C*-spiro analogue. In their hands, during the deprotection of the 1,2-diol and then the peracetylation only the pyranose glycone moiety was obtained. In order to obtain the target 3'-*C*-spiro nucleoside analogue **165**, the authors reported a new route using the same key reactions: formation of the diyne, *N*-glycosidation and [2+2+2]-cyclotrimerization [41]. Starting from D-xylose (**157**), the propargyl derivative **158** was obtained in five steps [42]. Propargylation of the alcohol **158** followed by a sequence of deprotection/protection of the primary hydroxyl group furnished the pivaloyl ester **161** in 66% yield (three steps). Selective acetonide hydrolysis of **161** and peracetylation gave an anomeric mixture of diacetates **162** in 87% yield (two steps). Conventional Vorbrüggen methodology followed by Zemplen’s deacylation permitted to obtain selectively the corresponding nucleoside analogue **164** as platform molecule for the cyclotrimerization in 58% yield (two steps). Using a similar protocol described above [38,39], the substitution of the Wilkinson catalyst by Cp*RuCl(cod) (Ru *vs.* Rh) permitted to prepare the target 3'-*C*-spiro nucleoside analogue **165** in 79% yield (Scheme 20).

*Reagents and Conditions*: (i) Reference [42]; (ii) NaH, propargyl bromide, THF, 0 °C to rt, 3 h, 83%; (iii) TBAF, THF, rt, 8 h, 98%; (iv) PivCl, TEA, DMAP, CH_2_Cl_2_, 0 °C to rt, 6 h, 81%; (v) (a) Aq AcOH (60%), reflux, 2 h; (b) Ac_2_O, TEA, DMAP, CH_2_Cl_2_, 87% for the two steps; (vi) uracil, BSA, TMSOTf, CH_3_CN, 50 °C, 2 h, 75%; (vii) MeONa, MeOH, rt, 20 min, 78%; (viii) Cp_*_RuCl(cod) (5 mol %), C_2_H_4_Cl_2_, EtOH, rt, 4–6 h, 79%.

**Figure 9:**
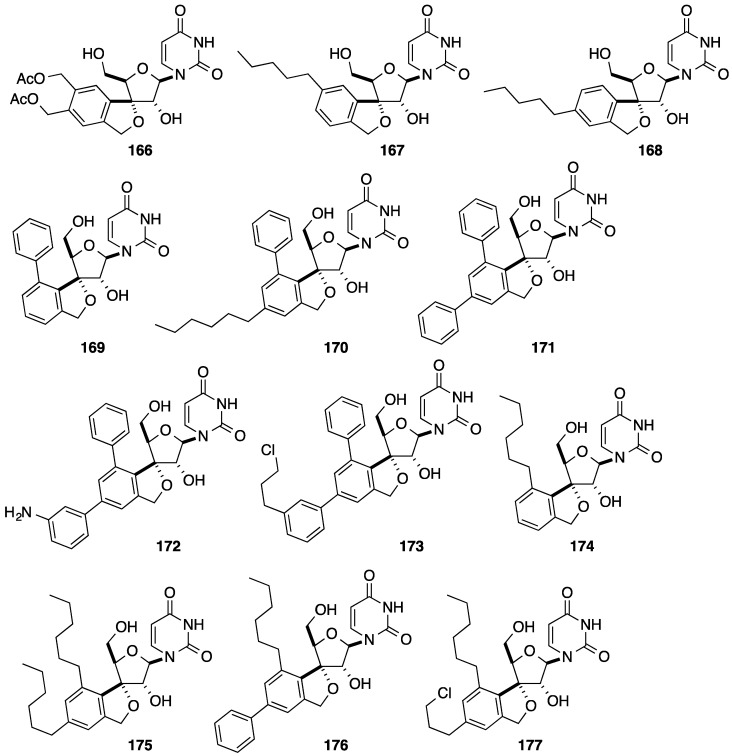
3'-*C*-Spiro uridine analogues **166**–**177**.

By using symmetric and unsymmetric alkynes, application of this strategy permitted different 3'-*C*-spiro nucleoside analogues **166**–**177** having substituted phenyl core to be obtained (Figure 9) [41].

## 6. Construction of the Glycone Part Starting from an Aromatic Moiety

The synthesis of nucleoside analogues having a *C*-*C* bond between an aromatic core and the glycone moiety can be realized starting from a benzene derivative via a multi-step strategy. In this regards, chloroacetophenone and benzaldehyde derivatives were used as starting materials.

**Scheme 21:**
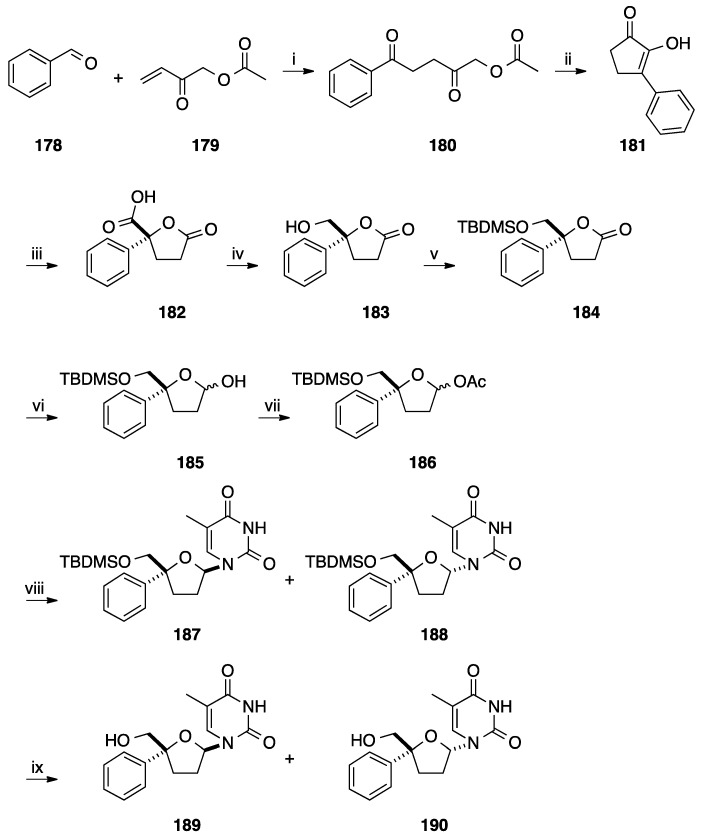
Synthesis of the 4'-*C*-phenyl thimidine analogues **189** and **190**.

In 2009, Lopp and co-workers described the enantioselective synthesis of 4'-aryl-2',3'-dideoxy-nucleoside analogues in nine steps (Scheme 21) [43]. Starting from benzaldehyde (**178**), addition of the 1-acetoxybut-3-en-2-one (**179**) furnished the corresponding ester **180** and then treatment in basic media gave the corresponding lactone **181** in 28% yield (two steps). Enantioselective oxidation of the enol tautomer **181** was realized in presence Ti(O*i*-Pr)_4_, *t*-BuOOH and (+)-diethyl tartrate and permitted the preparation of the carboxylic acid **182** in 36% yield (ee 86%). It was notable that the formation of the keto acid was observed (16% yield) and a considerable amount of starting material **181** remained unreacted, permitting a recycling step [44]. With the chiral compound **182** having the D-configuration, Lopp and co-workers developed a conventional strategy for the preparation of the target nucleoside analogues **189**. Subsequent reduction of the carboxyl group of **182** using a borane complex, protection of the resulting hydroxyl group and reduction of the lactone furnished the two diastereoisomeric lactols **185** in 79% yield (three step). Then, acetylation followed by *N*-glycosidation and deprotection of the primary hydroxyl group gave, after flash chromatography, the target β-D-isomer **189** and α-D-isomer **190** in 40% yield, respectively.

*Reagents and Conditions*: (i) 3-Benzyl-5-(2-hydroxyethyl)-4-methylthiazolium chloride, TEA, dioxane, 70–80 °C, 15 h, 71%; (ii) MeONa, MeOH, reflux, 40%; (iii) Ti(O*i*-Pr)_4_, (+)-diethyl tartrate, *t*-BuOOH, CH_2_Cl_2_, −20 °C, 114 h, 36% (ee 86%); (iv) BH_3_.SMe_2_, THF, 86%; (v) TBDMSCl, imidazole, CH_2_Cl_2_, 100%; (vi) DIBAL-H, toluene, 92%; (vii) Ac_2_O, TEA, CH_2_Cl_2_, 90%; (viii) thymine, BSA, TMSOTf, CH_3_CN, 88% (**187**:44%, **188**:44%); (ix) TBAF, THF, 100%.

**Figure 10:**
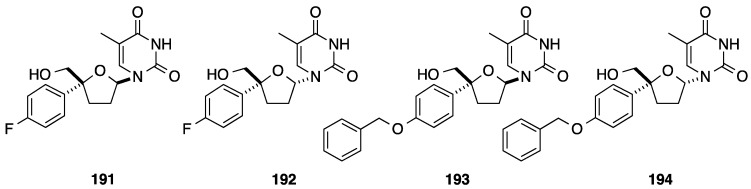
4'-*C*-Phenyl thimidine analogues **191**–**194**.

Lopp and co-workers developed in the same paper the formation of substituted benzene derivatives **191**–**194** (Figure 10) [43].

**Scheme 22:**
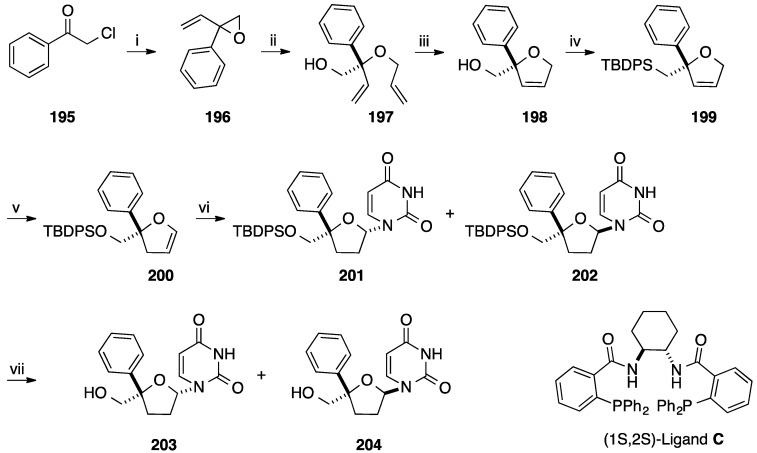
Synthesis of the 4'-*C*-phenyl L-uridine analogues **203** and **204**.

In 2003, Trost and co-workers published a elegant synthesis of 4'-*C*-phenyl nucleoside analogues as depicted in Scheme 22. The authors reported a multi-step strategy using the Pd-catalyzed dynamic kinetic asymmetric transformation (Pd-DYKAT) of vinylepoxide, metathesis, isomerization and *N*-glycosidation [45]. Starting from the chloroacetophenone (**195**) addition of vinylmagnesium bromide furnished the phenyl substrate **196** in 91% yield. The racemic mixture of **196** reacted with allylic alcohol in presence of Pd_2_(dba)_3_.CHCl_3_ as pre-catalyst, (1*S*,2*S*)- diphosphine **C** as chiral ligand and triallyl borate as co-catalyst to give the 2-(*S*)-phenyl-2-allyloxybut-3-en-1-ol (**197**) in 33% yield (ee 87%). Ring-closing metathesis was realized with Grubbs I catalyst (2 mol %) and furnished the desired compound **198** in 86% yield. In order to allow the nucleoside base installation, protection of the primary hydroxyl group and then isomerization to convert 2,5-dihydrofuran **199** to 2,3-dihydrofuran **200** were realized. The authors obtained the glycal **200** in 90% yield using [H_2_Ru(CO)(PPh_3_)_3_] as ruthenium catalyst. The *N*-glycosidation was effected using the silylated uracil and PhSeCl in presence of InCl_3_ as Lewis acid and gave a mixture of the two anomeric products **201** and **202** in 67% yield. The major nucleoside analogue **201** had the β-L-configuration which is the enantiomeric form of the natural nucleoside. After classical treatment of **201** and **202** with TBAF and then purification by preparative HPLC, the deprotected 4'-*C*-phenyl β-L-isomer **203** and α-L-isomer **204** were obtained in 85% and 13% yields, respectively.

*Reagents and Conditions*: (i) CH_2_CHMgBr, THF, 91%; (ii) [Pd_2_dba_3_].CHCl_3_, (1*S*,2*S*)- Ligand **C**, DMAP, dioxane, (CH_2_CHCH_2_)_3_B, rt, 19 h, 33% (ee 87%); (iii) Cl_2_(PCy_3_)_2_RuCHPh, CH_2_Cl_2_, rt, 11 h, 86%; (iv)TBDPSCl, imidazole, DMAP, DMF, rt, 45 mn, 97%; (v) [H_2_Ru(CO)-(PPh_3_)_3_], toluene, rt, 70 °C, 4 h and then 80 °C, 9 h, 90%; (vi) PhSeCl, dioxane, bis(trimethylsilyl)uracil, InCl_3_, rt, 3 h, 67%; (vii) TBAF, THF, rt, 1.5 h, **203**:85%, **204**:13%.

**Figure 11:**
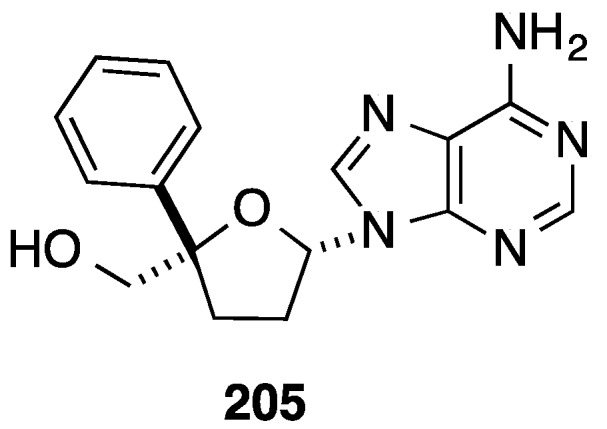
4'-*C*-Phenyl purine analogue **205**.

In the same paper and using the same methodology, Trost and co-workers described the synthesis of the purine analogue **205** (Figure 11) [45].

**Scheme 23:**
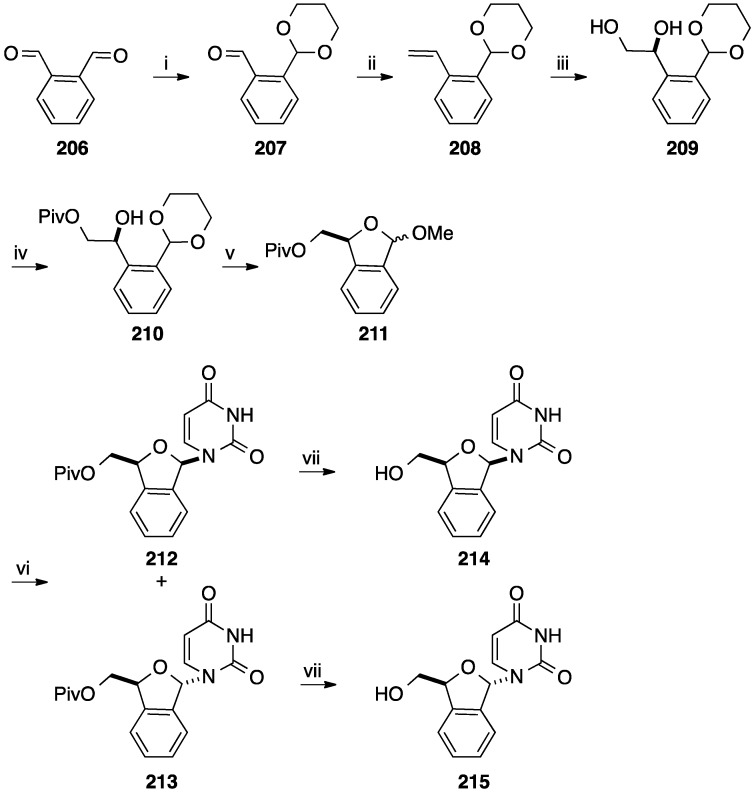
Synthesis of the benzo[*c*]furan analogues of d4U **214** and **215**.

*Reagents and Conditions*: (i) propan-1,3-diol, PTSA, toluene, 110 °C, 5 h, 75%; (ii) Ph_3_PCH_2_Br, *n*-BuLi, THF, 0 °C to rt, 1 h, 77%; (iii) AD-mix α, aq. *t*-BuOH, −10 °C to 0 °C, 1 h, 85% (ee > 99%); (iv) PivCl, TEA, toluene, −10 °C, 12 h, 78%; (v) MeOH, HCl, rt, 1 h, 72%; (vi) silylated uracil, SnCl_4_, C_2_H_4_Cl_2_, 0 °C, 2 h, **212**:35%, **213**:38%; (vii) aq. NaOH (1N), rt, 2 h, 93%.

**Figure 12:**
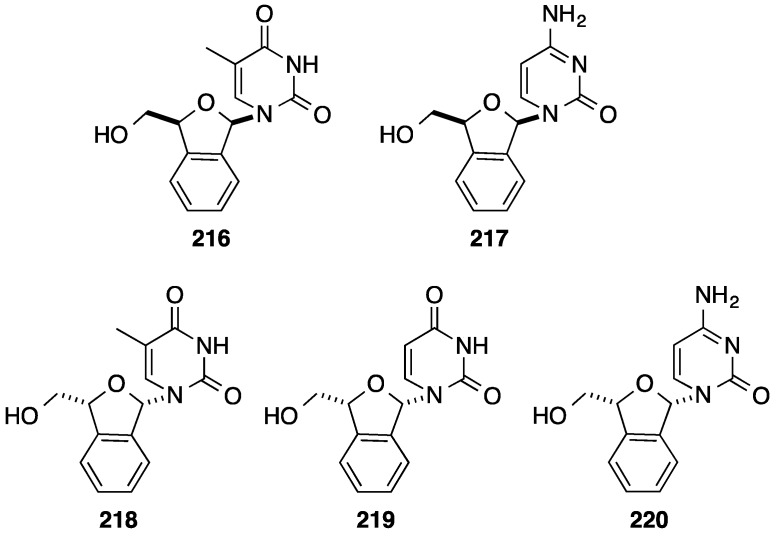
Benzo[*c*]furan nucleoside analogues **216**–**220**.

From 1996 to 2006 Len and co-workers described the synthesis of d4T analogues having a benzo[*c*]furan core. Various strategies including racemic synthesis and asymmetric synthesis have been reported by his group, all of them starting from phthalaldehyde [46,47,48,49,50,51,52,53,54,55]. In parallel to this work, the Liu group reported the racemic synthesis of benzo[*c*]furan nucleoside analogues [56]. For simplicity, only the asymmetric synthesis of benzo[*c*]furan analogues reported by Len is described here [48]. After selective protection of phthalaldehyde **206**, Wittig homologation of the remaining formyl group gave the corresponding styrene **208** in 58% yield (Scheme 23). Asymmetric dihydroxylation of the vinyl group using the commercial Sharpless reagent, AD-mix α afforded the corresponding dihydro derivative **209** in 85% yield (ee > 99%). The enantioselectivity of the dihydroxylation was important since only the new stereocenter having *S*-configuration can furnish the D-series. In order to avoid the isochroman formation, selective benzoylation of the primary hydroxyl group of **209** was necessary. Then classical treatment of the ester **210** in acidic methanol permitted the deprotection of the formyl group, the cyclization and methylation to afford a mixture of the two anomeric 1,3-dihydrobenzo[*c*]furan derivatives **211** in 82% yield. Without separation of the two epimers **211**, standard Vorbüggen chemistry furnished the β-isomer **212** and α-isomer **213**, due to the lack of neighboring group participation to direct stereoselectivity. After removal of the benzoyl protection and subsequent silica gel chromatography, the target nucleosides **214** and **215** were obtained enantiomerically pure in 19% and 9% overall yield, respectively. The related enantiomers analogous to L-nucleosides were synthesized using the same strategy but employing AD-mix β. Using the same strategy, Len and co-workers [46,47,48,49,50,51,52,53,54,55] reported the synthesis of d4T analogues **216**–**220** (Figure 12).

## 7. Conclusions

Different syntheses of *C*-aryl nucleoside analogues have been driven by attempts to improve upon the biological activities of commercial antiviral and antitumoral nucleosides, to provide structure–activity data and to offer a continuity of new drugs as alternatives to the previous generation to combat the rise of resistance. Formation of *C*-aryl nucleoside analogues using three main strategies depending of the starting materials—nucleoside, carbohydrate or aromatic compounds—was described.

Starting from nucleoside analogues, addition of an aromatic ring on a keto group or an epoxide and *C*-*C* cross coupling were reported. First, classical oxidation of the glycone part and then addition of phenyllithium or triphenylaluminium permitted preparation of 2'-*C*, 3'-*C* and 5'-*C*-phenyl derivatives [8,11,22]. Selective addition of triphenylaluminium on an nucleoside analogue having an epoxide in position 1',2' was also reported [35]. *C*-*C* cross coupling was described for the formation of 2'-*C*- and 3'-*C*-aryl-2',3'-didehydro-2',3'-dideoxynucleoside analogues using two multi-step strategies: palladium-catalyzed cross coupling via bromovinyl nucleoside analogues [26,27] and palladium-catalyzed cross coupling via butyltinvinyl nucleoside analogues [29]. A more efficient short strategy was reported by the same group starting from d4T and d4U with the formation of the 3'-*C*-phenyl derivative via the corresponding butyltinvinyl nucleoside analogues [33,34]. It was noteworthy that 3'-*C*-phenyl d4U derivatives were used as intermediate for the formation of the corresponding ddC analogues by conventional hydrogenation [34].

Starting from carbohydrate analogues, two strategies were reported: addition of aromatic ring on a keto group and [2+2+2]-cyclotrimerization. Starting from ribose, addition of phenyllithium on a 1,4-ribonolactone derivative followed by a *N*-glycosidation allowed the synthesis of the corresponding 1-*C*-phenyl nucleoside analogues and the corresponding bicyclo derivatives [13,14,15,16,17]. Another strategy was developed for the preparation of 4'-*C*-phenyl nucleoside analogues using addition of phenyllithium or phenylmagnesium bromide on a lactol followed by *N*-glycosidation [19]. Tricyclonucleoside analogues were obtained in a multi-steps strategy starting from D-glucose and D-xylose. The main key reactions were the formation of the corresponding diyne, the [2+2+2]-cyclotrimerization and then the *N*-glycosidation. This strategy permitted to prepare different isochromane analogues having a bridge between the 3'-*C* and 4'-*C* carbon atoms [38,39]. A modified strategy using the [2+2+2]-cyclotrimerization at the end of the protocol afforded the 3'-*C*-spiro-annulated nucleoside analogues [41].

Different approaches were reported starting from achiral compounds having an aromatic core or not. In these cases, asymmetric syntheses were studied. Most often the 4'-*C*-phenyl nucleoside analogues were obtained using this strategy. Starting from achiral 1,3-dihydroxyacetone derivative, asymmetric α-alkylation was studied and then diastereoselective addition of phenylmagnesium bromide permitted introduction of the aromatic ring on the skeleton [21]. Then classical glycone formation followed by *N*-glycosidation furnished the target nucleoside analogues. Starting from benzaldehyde, the enol intermediate was used for enantioselective oxidation and then the corresponding lactone afforded the 4'-*C*-phenyl nucleoside analogues using a classical protocol [43,44]. Chloroacetophenone was used as starting material for the synthesis of 4'-*C*-phenyl L-nucleosides as enantiomers of the natural nucleoside series [45]. The key reaction used was a Pd-catalyzed dynamic kinetic asymmetric transformation furnishing the enantiomeric diene. Then through classical ring closing metathesis, isomerization and *N*-glycosidation, the target nucleoside analogues were obtained. The 2'-*C*-phenyl nucleoside analogues were obtained starting from glyceraldehyde derivatives. Asymmetric *Si*-face addition of allyltitanium reagent furnished diastereoselectively the corresponding alcohol as key intermediate. Then conventional formation of the glycone moiety followed by *N*-glycosidation furnished the target nucleoside analogue. Finally, a series of 2'-*C*- and 3'-*C*-dibranched nucleosides with a benzo[*c*]furan core have been synthesised by a convergent route employing conventional Vorbruggen chemistry on a preformed benzo[*c*]furan system [48]. An interesting feature of the route to this unusual glycone system in nucleoside chemistry is the highly effective use of the stereoselective Sharpless hydroxylation to obtain compounds analogous to conventional nucleosides in the D- and L-series, accordingly.
